# Hippo pathway inhibition promotes metabolic adaptability and antioxidant response in myoblasts

**DOI:** 10.1038/s41598-023-29372-8

**Published:** 2023-02-08

**Authors:** Qi Liu, Su Pan, Pengyang Li, Richard A. F. Dixon

**Affiliations:** 1 Wafic Said Molecular Cardiology Research Laboratories, The Texas Heart Institute, MC 2-255, P.O. Box 20345, Houston, TX 77225-0345 USA; 2 Division of Cardiology, Pauley Heart Center, Virginia Commonwealth University, Richmond, VA USA

**Keywords:** Cell growth, Cell signalling

## Abstract

Metabolic plasticity in a hostile environment ensures cell survival. We investigated whether Hippo pathway inhibition contributed to cell adaptations under challenging conditions. We examined metabolic profiles and fuel substrate choices and preferences in C2C12 myoblasts after Hippo pathway inhibition via Salvador knockdown (SAV1 KD). SAV1 KD induced higher ATP production and a more energetic phenotype. Bioenergetic profiling showed enhanced key mitochondrial parameters including spare respiratory capacity. SAV1 KD cells showed markedly elevated glycolysis and glycolytic reserves; blocking other fuel-oxidation pathways enhanced mitochondrial flexibility of glucose oxidation. Under limited glucose, endogenous fatty acid oxidation increased to cope with bioenergetic stress. Gene expression patterns after SAV1 KD suggested transcriptional upregulation of key metabolic network regulators to promote energy production and free radical scavenging that may prevent impaired lipid and glucose metabolism. In SAV1 KD cells, sirtuin signaling was the top enriched canonical pathway linked with enhanced mitochondrial ATP production. Collectively, we demonstrated that Hippo pathway inhibition in SAV1 KD cells induces multiple metabolic properties, including enhancing mitochondrial spare respiratory capacity or glycolytic reserve to cope with stress and upregulating metabolic pathways supporting elevated ATP demand, bioenergetics, and glycolysis and counteracting oxidative stress. In response to metabolic challenges, SAV1 KD cells can increase fatty acid oxidation or glucose-coupled oxidative phosphorylation capacity to compensate for substrate limitations or alternative fuel oxidation pathway inhibition.

## Introduction

Energy metabolism is central to cell function. Carbohydrates, amino acids, and fats provide fuel to generate adenosine triphosphate (ATP), which is required for cell growth and survival^1^. Oxidative phosphorylation (OXPHOS) and glycolysis are two major metabolic pathways for ATP production. OXPHOS uses intermediate metabolites from the tricarboxylic acid cycle to synthesize ATP via electron transport in mitochondria^2–4^ and is a main energy source of cells in the presence of oxygen. Glycolysis is an inefficient way to produce ATP (2 molecules/molecule of glucose) but is oxygen independent and has a faster ATP production rate. Additionally, glycolysis produces pyruvate, which drives ATP production via OXPHOS^5,6^. Therefore, OXPHOS and glycolysis are intertwined and cooperate to maintain the cell’s energy balance. In harsh environments, cells with increased metabolic flexibility in using fuels available for ATP production survive better than cells that are metabolically rigid.

Mitochondrial respiration controls ATP generation and serves as a main source of reactive oxygen species (ROS) production^7–9^. Spare respiratory capacity (SRC) is the difference between basal and maximal respiration and determines the mitochondrial reserve for providing additional ATP under energy deficiency (ie, caloric restriction)^10^. The reduction of SRC, which measures the ability of the cell to survive, increases cell vulnerability during an energy crisis. As a key parameter for quantifying mitochondrial fitness, the SRC depends on mitochondrial bioenergetics, which are regulated by a complex network of pathways and key signaling molecules such as sirtuin signaling and transcriptional coactivator peroxisome proliferator-activated receptor γ (PPARγ) coactivator 1α (PGC-1α)^11,12^. As a consequence of mitochondrial respiration, ROS are generated by a series of redox reactions during electron transport^9^. Increased ROS levels may augment intracellular oxidative stress to cause cell damage^8^. To combat ROS accumulation, ROS scavengers (e.g., catalase, superoxide dismutase, glutathione peroxidase) have cell detoxifying capabilities that reduce ROS-induced injury^13^.

The ability of skeletal muscle cells to efficiently select fuel or metabolic programs in response to environmental stimuli and energy demand is referred to as metabolic flexibility^14^. This flexibility plays a crucial role in cell proliferation and survival and muscle regeneration under adverse conditions. Myoblasts are myogenic progenitor cells that are derived from activated muscle stem cells (satellite cells) and are responsible for multinuclear myotube formation during skeletal muscle growth^15,16^. The proliferation of myoblasts requires synthesis of proteins, lipids, and nucleic acids. Therefore, these cells must be equipped with adaptive metabolic programs to sustain the elevated energy demand during biosynthetic processes of the cell cycle and to maintain energy homeostasis for survival under harsh conditions (e.g., nutrient scarcity, oxidative stress). Yet little is known about the molecular mechanisms underlying the regulation of the metabolic adaptability of myoblasts in response to environment cues. Specifically, these molecular mechanisms regulate mitochondrial bioenergetics and associated gene expression, glycolytic capacity, and fuel substrate selection for OXPHOS. Understanding how potential signaling pathways orchestrate energy production, mitochondrial respiration, and glycolytic function is critical to augment the efficiency of myoblast-induced muscle regeneration.

Hippo signaling is a highly conserved kinase cascade pathway^17^. As an adaptor protein and positive effector, Salvador (SAV1) physically interacts with MST1/2 kinases to activate LATS1/2 via phosphorylation. The activated LATS1/2 then phosphorylates the downstream transcriptional co-activator YAP, leading to its nucleus exclusion and Hippo pathway activation. SAV1 has been recognized as a key regulator of Hippo signaling in organ size control and skeletal muscle regeneration^18,19^. However, little is known about the effect of SAV1 on cellular metabolic pathways and bioenergetic function. Skeletal muscles are energy-demanding tissues, and the ability to efficiently adapt metabolic pathways in response to environmental changes would improve growth and survival benefits. Thus, we examined the ability of SAV1 to regulate fundamental bioenergetics and energy production in myoblasts. Here, we found that Hippo pathway inhibition by SAV1 knockdown (KD) in C2C12 myoblasts increased mitochondrial bioenergetics and cell survival. Compared with control cells, SAV1 KD cells showed significantly higher mitochondrial SRC and glycolytic capacity, had better adaptive responses to fuel availability, and were able to switch metabolic programs in response to environmental changes.

## Results

### Knockdown of SAV1 increases ATP production in C2C12 myoblasts

**Figure 1:**
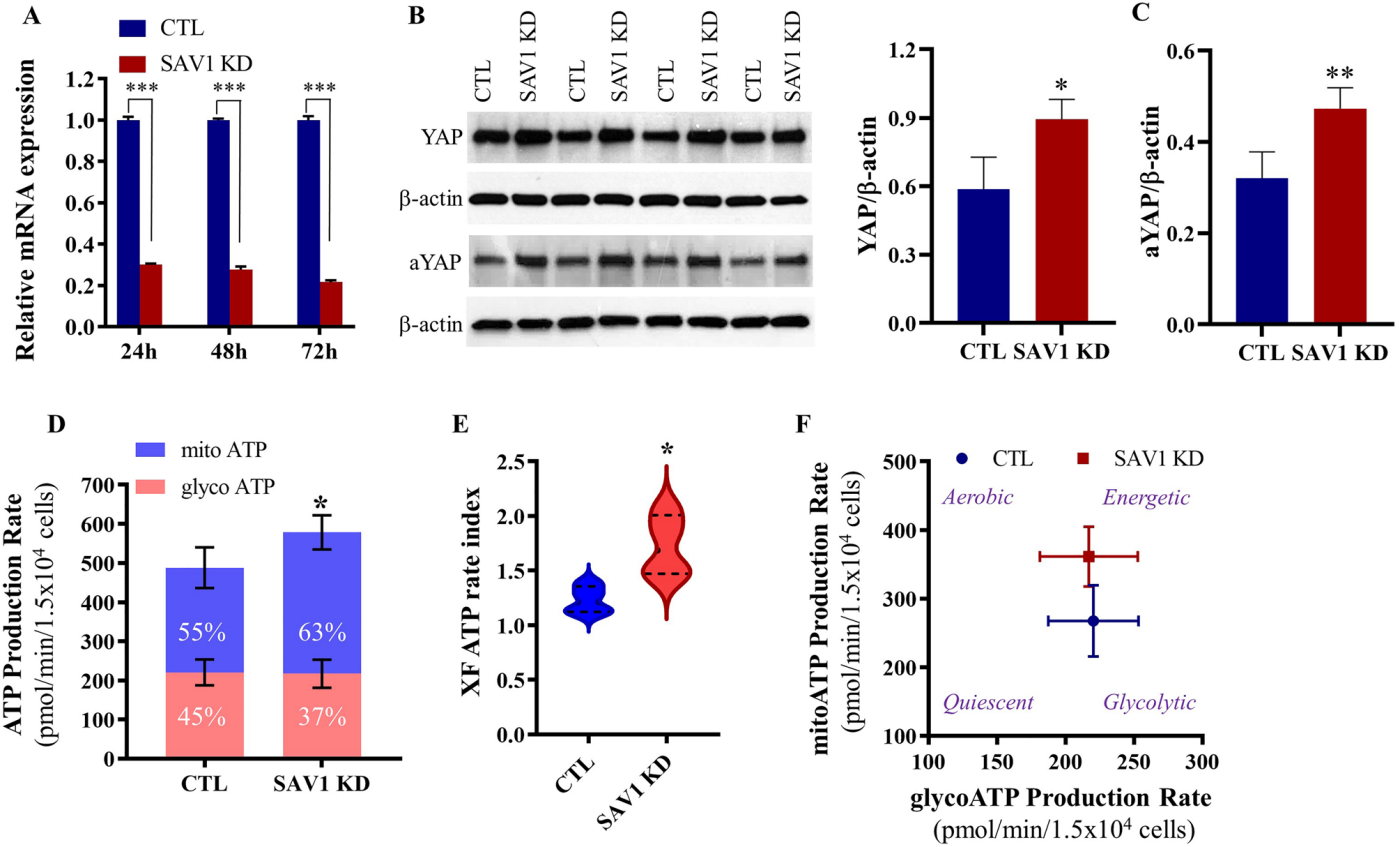
SAV1 KD in C2C12 myoblasts enhanced cell ATP production rate. (**A**) RT-qPCR indicates that the relative expression level of SAV1 mRNA was significantly suppressed after transfection of C2C12 myoblasts with SAV1 siRNA (SAV1 KD) at 24, 48, and 72 h as compared to negative control siRNA (CTL). Unpaired t test; data are mean ± SD; n = 3 independent assays; ***p < 0.001. (**B**) Representative western blot images show total YAP and the non-phosphorylated active form of YAP1 (aYAP) protein levels at 72 h after siRNA transfection. β-actin was used as a loading control. (**C**) Semiquantitative analysis shows a significant increase in YAP and aYAP in SAV1 KD cells compared with CTL cells. Unpaired t test; data are mean ± SD; n = 4; *p < 0.05, **p < 0.01. (**D**) Concurrent mitochondrial ATP and glycolytic ATP production rate in real-time under basal conditions. SAV1 KD cells showed a higher contribution of ATP production by mitochondrial oxidative phosphorylation than did CTL cells. (**E**) An increase of XF metabolic rate index (the ratio of mitoATP/glycol ATP) in SAV1 KD cells suggests a more oxidative phenotype than CTL cells. (**F**) Energetic map displays overall metabolic potential between SAV1 KD cells and CTL cells. Unpaired t test; data are mean ± SD; n = 4. *p < 0.05 (**D**,**E**).

To examine whether SAV1 affects cell energy metabolism, we used RNA interference to knockdown SAV1 in mouse C2C12 myoblasts, a well-established myogenic cell line for studying skeletal muscle function in vitro. The downregulation of SAV1 mRNA was confirmed by quantitative reverse transcription polymerase chain reaction (RT-qPCR) and semi-quantitative western blot analysis. In time course studies, compared to cells transfected with negative control siRNA (CTL), SAV1 siRNA transfected cells showed significantly lower levels of *Sav1* mRNA from 24 to 72 h. The lowest expression of *Sav1*mRNA was detected at 72 h post-transfection (1.000 ± 0.019 vs 0.216 ± 0.009, CTL vs SAV1 KD, Fig. 1A). SAV1 KD led to a significant increase in the Hippo pathway effector total YAP and the non-phosphorylated, active form of YAP1 (Fig. 1B,C; Supplementary Figs. 1–2), thus supporting inhibition of the Hippo pathway.

We performed a real-time ATP synthesis rate assay on the Seahorse extracellular flux analyzer, which measures the total ATP production rate in cells and distinguishes between ATP produced from mitochondrial OXPHOS (mito ATP) and glycolysis (glycol ATP). Although a combination of OXPHOS and glycolysis was actively used in both CTL and SAV1 KD cells, more than 50% of total ATP was generated through OXPHOS (55% and 63% of total ATP, respectively in Fig. 1D). The total ATP production rate was significantly higher in SAV1 KD cells than in CTL cells, and this increase occurred through increased mitochondrial OXPHOS metabolism (average 1.41-fold increase of mitoATP). The ATP rate index (a ratio of mitoATP production rate to glycoATP production rate) indicates SAV1 KD shifted cells to a more oxidative phenotype (Fig. 1E). The energy map suggests that SAV1 KD cells had a higher metabolic potential than CTL cells, indicating that SAV1 KD induced a more energetic state in myoblasts (Fig. 1F).

### Knockdown of SAV1 increases SRC and glycolytic reserve in response to energy demand

**Figure 2:**
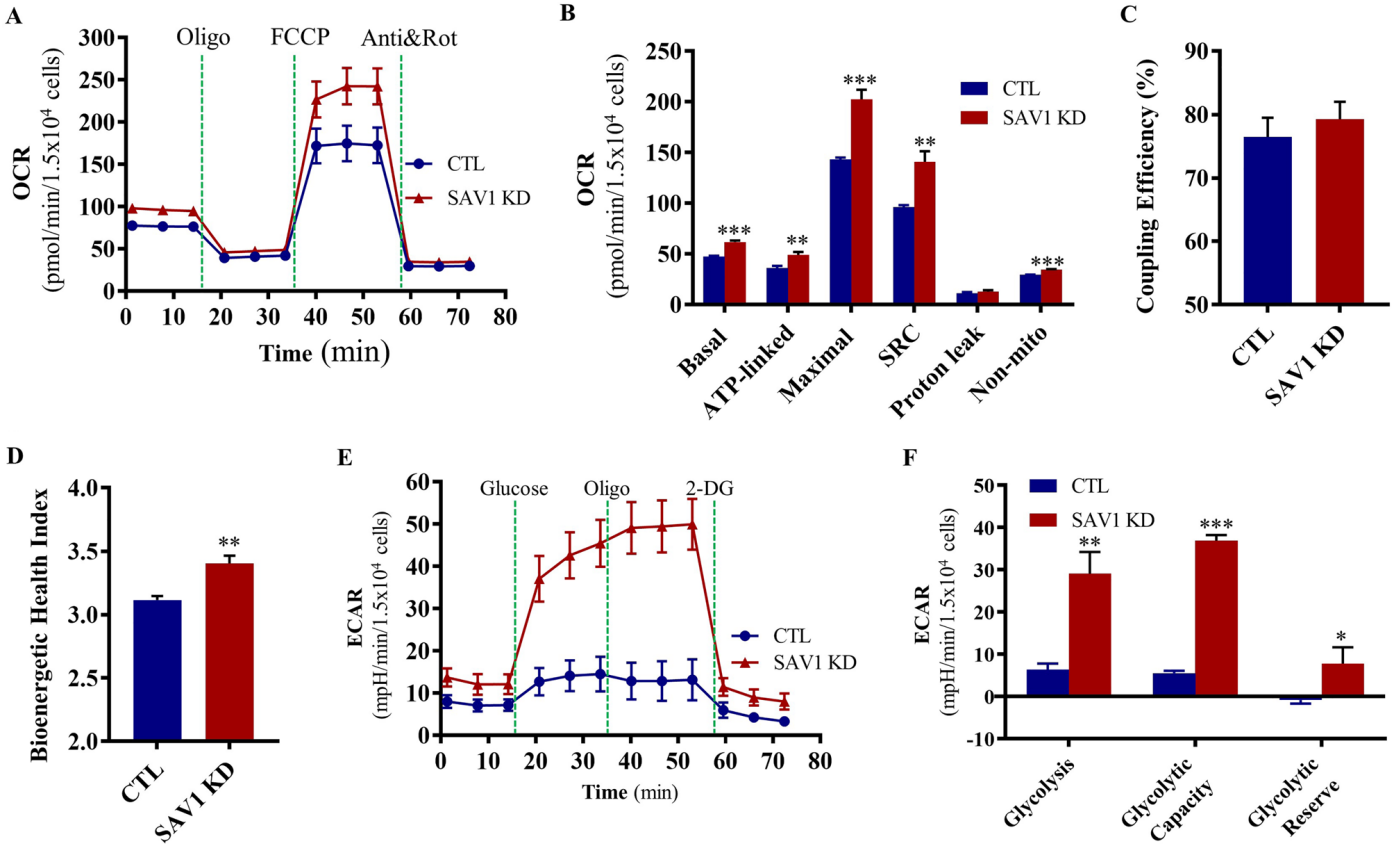
SAV1 KD in C2C12 myoblasts increased mitochondrial bioenergetics and glycolytic reserve to cope with stress. (**A**) Representative kinetic graph of oxygen consumption rate (OCR) measurements in the mitochondrial stress test with sequential injection of oxidative phosphorylation inhibitors as indicated. (**B**) SAV1 KD improved the key metabolic parameters of mitochondrial respiration accompanied with significant enhancement of spare respiratory capacity (SRC). ***p < 0.001, **p < 0.01. (**C**) Coupling efficiency was similar between CTL and SAV1 KD groups. (**D**) SAV1 KD significantly increased the mitochondrial bioenergetic health index. **p < 0.01. Unpaired t test; data are mean ± SD, n = 3 independent measurements (**B**–**D**). (**E**) Representative kinetic tracing of extracellular acidification rate (ECAR) measurements in glycolysis stress test with sequential compound injections as indicated. (**F**) SAV1 KD cells showed significant enhancement of glycolysis, glycolytic capacity, and reserve. Unpaired t test; data are mean ± SD, n = 3 independent measurements. ***p < 0.001, **p < 0.01, *p < 0.05.

To examine SAV1’s ability to modulate mitochondrial OXPHOS, we measured the cell oxygen consumption rate (OCR), which is an indicator of mitochondrial function, in real-time under a series of challenging conditions. SAV1 KD cells had a higher basal OCR than did control cells (1.5 × 10^4^ cells; 61.76 ± 1.56 vs 47.32 ± 0.82 pmol/min, SAV1 KD vs CTL; Fig. 2A,B). When cells were sequentially exposed to oligomycin (an ATP synthase inhibitor), FCCP (a protonophoric uncoupler), and rotenone/antimycin A (electron transport chain inhibitors) to induce mitochondrial stress, SAV1 KD significantly enhanced ATP-linked OCR (48.98 ± 2.92 vs 36.19 ± 2.06 pmol/min), maximal OCR (202.70 ± 9.26 vs 143.50 ± 1.56 pmol/min), and non-mitochondrial OCR (34.58 ± 0.44 vs 29.60 ± 0.16 pmol/min Fig. 2A,B). Proton leaks were similar in both cell groups. Notably, SAV1 KD resulted in a significant increase in SRC (the difference between maximal and basal OCR; 140.90 ± 10.52 vs 96.22 ± 1.98 pmol/min), indicating that mitochondria in SAV1 KD cells can achieve a higher rate of OXPHOS-dependent respiration and were better equipped to handle a surge in ATP demand if needed. Coupling efficiency, a measurement of the OCR fraction from basal respiration used for ATP synthesis, was similar in both groups (Fig. 2C). SAV1 KD cells had a significantly higher bioenergetic health index than did control cells (3.40 ± 0.06 vs 3.11 ± 0.03, Fig. 2D), suggesting increases in SRC and OCR linked to ATP production contribute to enhanced mitochondrial health.

We examined glycolytic function by measuring the real-time extracellular acidification rate (ECAR), which is an indicator of glycolysis, in CTL and SAV1 KD cells. Glucose, oligomycin, and 2-DG (a glucose analog that inhibits glycolysis) were sequentially injected into cells that were maintained in a basal medium containing only 2 mM of glutamine. The glucose-induced increase in ECAR is defined as glycolysis, as it reflects the breakdown of glucose into pyruvate. SAV1 KD induced a significant increase in glycolysis (1.5 × 10^4^ cells; 29.07 ± 5.17 vs 6.36 ± 1.46 mpH/min, Fig. 2E,F). When cells were treated with OXPHOS-disrupting oligomycin, the ECAR (glycolytic capacity) was substantially higher in SAV1 KD cells than in CTL cells (36.88 ± 1.33 vs 5.54 ± 0.61 mpH/min, respectively). Importantly, glycolytic reserve, the difference between glycolytic capacity and glycolysis rate, was significantly higher in SAV1 KD cells than in CTL cells (7.81 ± 3.85 vs −0.82 ± 0.85 mpH/min, respectively). Together, these data suggest that SAV1 KD can increase OXPHOS as well as glycolysis to help cells meet bioenergy demands. Moreover, SAV1 KD cells had higher mitochondrial SRC and glycolytic reserve to better endure stress than did CTL cells.

### Knockdown of SAV1 enhances cell proliferation and cell survival under H_2_O_2_-induced oxidative stress

**Figure 3:**
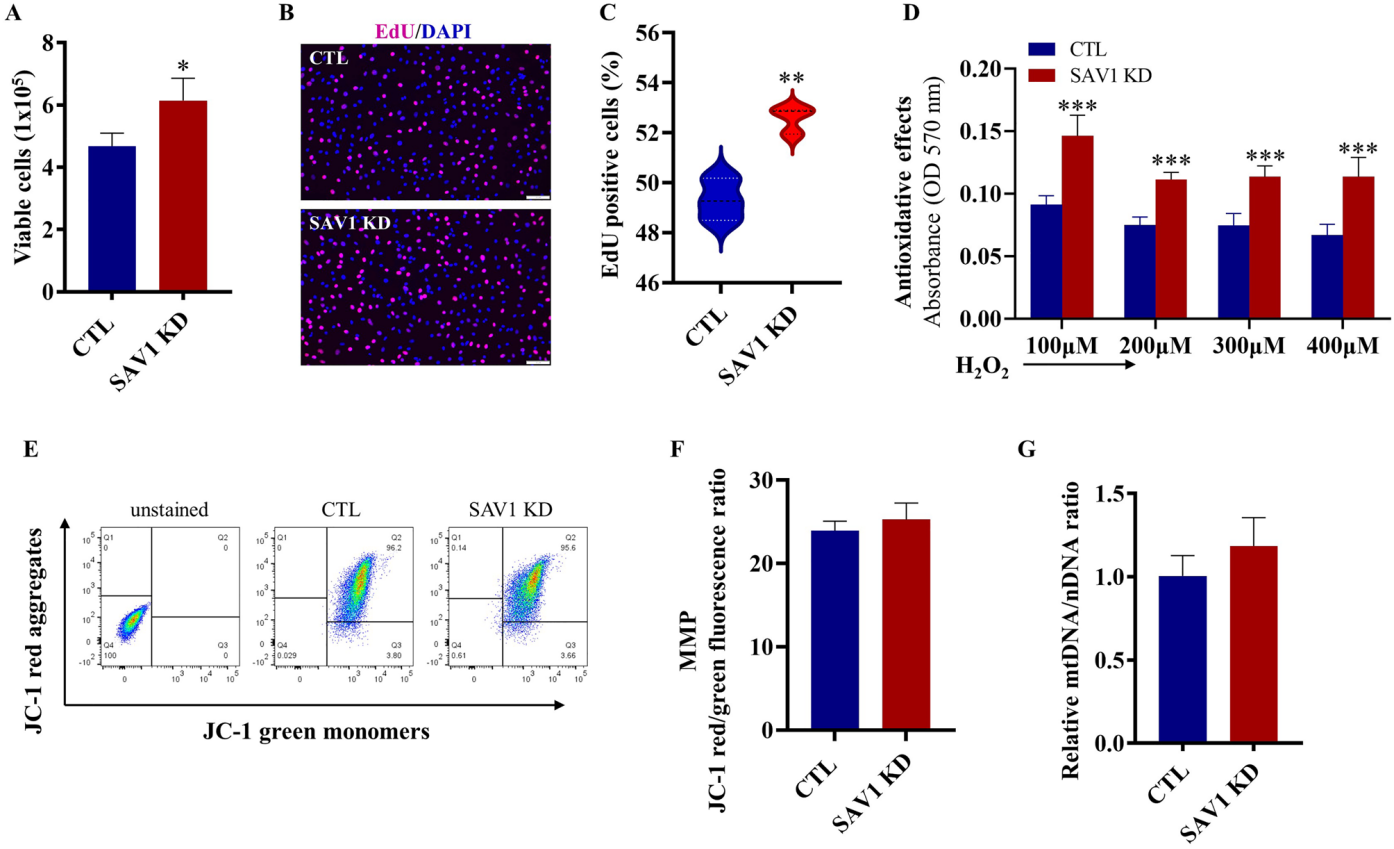
SAV1 KD in C2C12 myoblasts increased cell proliferation and survival. (**A**) Summary of viable cell counts with trypan blue exclusion in SAV1 KD cells and CTL cells at 48 h after SAV1 siRNA or negative control siRNA transfection. Data are mean ± SD; n = 4 independent counts. Unpaired t test; *p < 0.05. (**B**) Representative images of EdU-labeled nuclei in CTL and SAV1 KD cells at 72 h post-siRNA transfection. Cells were treated with EdU for 2 h before fixation. (**C**) SAV1 KD cells exhibited a higher percentage of EdU incorporation (EdU^+^ nuclei) than did CTL cells. Unpaired t test; data are mean ± SD; n = 3 independent assays. **p < 0.01. (**D**) MTT cell viability assay showed the significantly increased survival capability of SAV1 KD cells as compared to CTL cells after being exposed to 100–400 μM H_2_O_2_ for 2 h. Unpaired t test; data are mean ± SD, n = 3 independent assays. ***p < 0.001. (**E**) Flow cytometry analysis of mitochondrial membrane potential of CTL and SAV1 KD cells by JC-1 staining. (**F**) The ratio of cell percentage of JC-1 red fluorescent aggregates (percentage of cells in Q2) to cell percentage of JC-1 green fluorescent monomers (percentage of cells in Q3) is similar in CTL and SAV1 KD groups. Data are mean ± SD; n = 4 independent assays. (**G**) Analysis of mitochondrial DNA (mtDNA) content in SAV1 KD and CTL cells by quantitative PCR. *Nd1*(Mitochondrially Encoded NADH:Ubiquinone Oxidoreductase Core Subunit 1) and *Hk* (Hexokinase-2) genes were amplified and used to quantify the mtDNA/nDNA ratio. Data are mean ± SD; n = 3 independent assays.

At 48 h after SAV1 siRNA transfection into C2C12 myoblasts, viable cell numbers were higher in SAV1 KD cells than in CTL cells (6.15 ± 0.71 × 10^5^ vs 4.68 ± 0.41 × 10^5^, SAV1 KD vs CTL, respectively; Fig. 3A). To examine the effect of SAV1 KD in myoblasts on cell proliferation, we treated cells with the nucleotide analog 5-ethynyl-2′-deoxyuridine (EdU) for 2 h to label actively replicating DNA before cell fixation. At 72 h after SAV1 siRNA transfection, the percentage of EdU^+^ nuclei was significantly higher in SAV1 KD cells than in CTL cells (52.37 ± 0.31% vs 49.32 ± 0.49%; Fig. 3B,C). Because viable cells contain a mitochondrial dehydrogenase that catalyzes a color change by reducing tetrazolium compound MTT (3-(4,5-Dimethylthiazol 2-yl)-2,5-diphenyltetrazolium bromide), we used an MTT colorimetric assay to evaluate cell survival. At 2 h after treatment with H_2_O_2_ at various concentrations (100 to 400 µM), SAV1 KD cells were more metabolically active than CTL cells, reflecting an enhanced cell survival capability under H_2_O_2_-mimicked oxidative stress (Fig. 3D).

We assessed the mitochondrial membrane potential (MMP) of CTL and SAV1 KD cells by using the membrane-permeant JC-1 cationic fluorescent dye to stain mitochondria. Flow cytometry analysis was performed to quantify the percentage of cells with high MMP (forming red fluorescent J- aggregates) and low MMP (forming green fluorescent J-monomers). The ratio of cells (Q2) with red fluorescent J-aggregates to those (Q3) with green fluorescent J-monomers was used to monitor the change of MMP. Both CTL and SAV1 KD cells had a similar JC-1 aggregate/monomer ratio, reflecting a similar MMP when cultured in myoblast growth medium (Fig. 3E,F). Given that mitochondria contain multiple copies of DNA involved in cellular bioenergetic metabolism and redox homeostasis, we examined the ability of SAV1 KD to alter the mitochondrial DNA (mtDNA) content of cells. There was no marked difference in the mtDNA/nuclear DNA (nDNA) ratio between SAV1 KD and CTL cells (Fig. 3G).

### Knockdown of SAV1 regulates the gene network involved in energy metabolism and antioxidant defense

**Figure 4:**
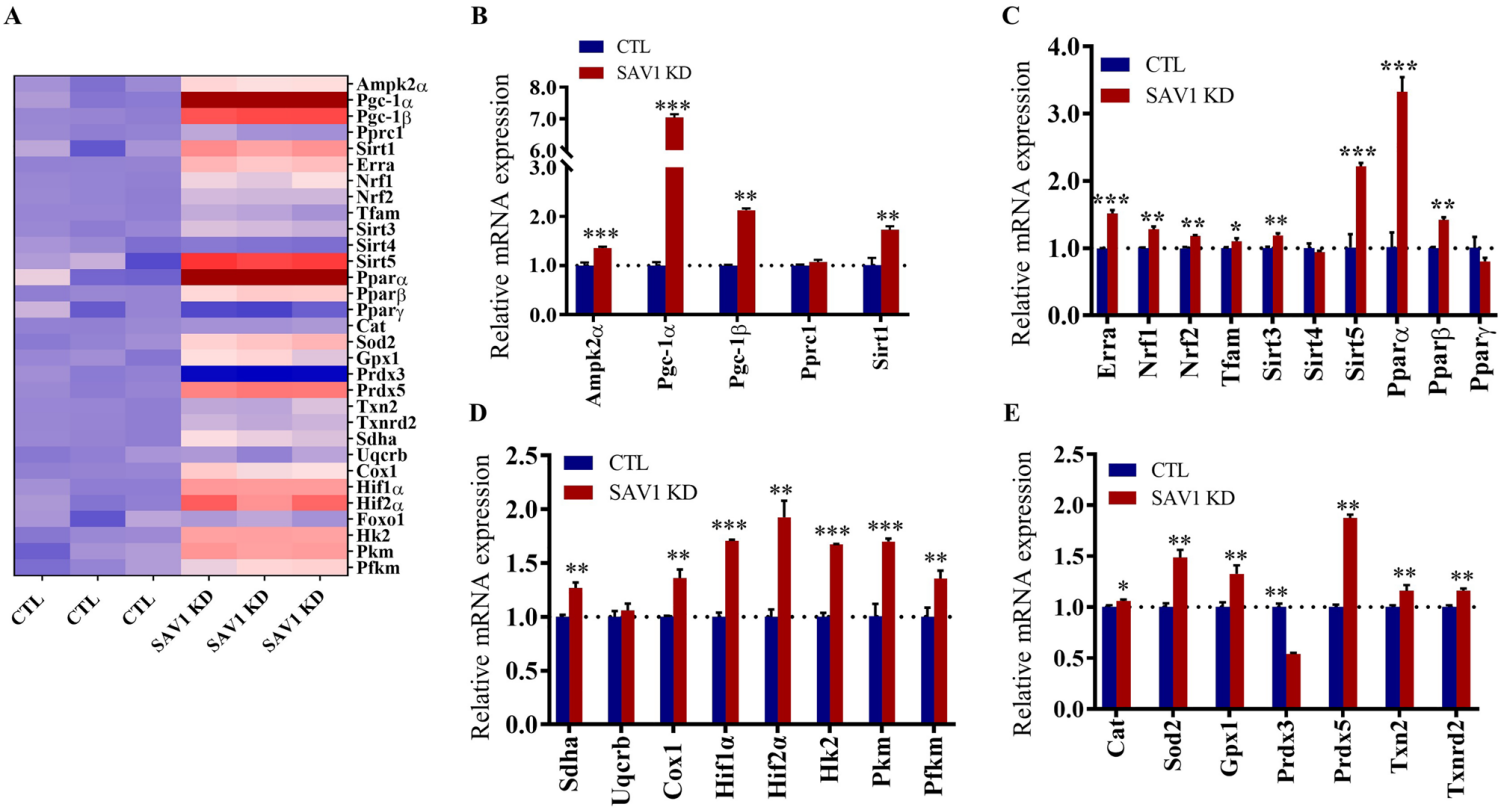
Quantitative analysis of gene expression profiles of SAV1 KD myoblasts. (**A**) Heat map visualization of real-time quantitative RT-PCR results showing the relative mRNA levels in SAV1 KD and CTL cells. The red color indicates high levels and the blue color indicates low levels of expression. (**B**–**E**) SAV1 KD significantly enhanced the transcription of genes regulating mitochondrial biogenesis, glycolysis, redox signaling, and the anti-oxidative response. Full gene names are described in the Results section. mRNA expression was normalized to 18 s rRNA. Unpaired t test; data are mean ± SD, n = 3 independent assays. ***p < 0.001, **p < 0.01, *p < 0.05.

We used quantitative RT-PCR to examine the transcriptional changes in an array of genes involved in energy production and expenditure after silencing SAV1 (Fig. 4A). Because AMP-activated protein kinase (AMPK), sirtuin 1 (SIRT1), and peroxisome proliferator-activated receptor γ (PPARγ) coactivator 1α (PGC-1α) together with PGC-1β and PGC-related co-activator (PPRC1) are linked to regulatory networks of metabolism, we quantified their mRNA levels 48 h after SAV1 siRNA transfection. Silencing SAV1 significantly increased transcript levels of *Ampk1α, Pgc-1α, Pgc-1β,* and *Sirt1* (*P* < 0.01, Fig. 4B). Among the genes tested, *Pgc-1α* was most affected by SAV1 KD, as its relative mRNA level was sevenfold higher in SAV1 KD cells than in CTL cells (7.03 ± 0.06 vs 1.00 ± 0.04, respectively).

PGC-1α is a transcriptional coactivator and master regulator of mitochondrial biogenesis^20^. It interacts with a broad array of transcription factors, including the PPAR family (PPARα, PPARβ, and PPARγ), the estrogen-related receptor α (ERRα), and nuclear respiratory factors 1 and 2 (NRF-1, NRF2) to enhance energy metabolism, which further regulates the transcription of nuclear-encoded mitochondrial genes such as mitochondrial transcription factor A (TFAM)^21^. RT-qPCR analysis revealed that mRNA levels of *Errα, Nrf1, Nrf2, Tfam, Pparα,* and *Pparβ* were significantly higher in SAV1 KD cells than in CTL cells (*P* < 0.01, Fig. 4C). In addition, the mRNA levels of other cellular metabolism-related genes were significantly increased by SAV1 KD in C2C12 myoblasts, such as mitochondrial sirtuins (*Sirt 3* and *Sirt 5*); transcriptional activator of glycolytic enzymes hypoxia inducible factor 1α (*Hif1α*) and *Hif2α*^22^; key enzymes that promote glycolysis such as hexokinase (*Hk2*), pyruvate kinase (*Pkm*), and phosphofructokinase (*Pfkm*)^23^; and a major catalytic subunit of succinate-ubiquinone oxidoreductase (*Sdha*) and cytochrome c oxidase (*Cox1*), complex II and complex IV of the mitochondrial respiratory electron transport chain (Fig. 4D).

Mitochondrial ATP generation by OXPHOS in the electron transport chain is the major source of ROS generation^24^. To examine the effect of SAV1 silencing on ROS defense systems, we quantified the mRNA expression of glutathione peroxidase-1 (*Gpx1*) and mitochondrial antioxidant and redox genes including manganese superoxide dismutase (*Sod2*), catalase (*Cat*), peroxiredoxin 3 (*Prdx3*) and 5 (*Prdx5*), thioredoxin 2 (*Txn2*), and thioredoxin reductase (*Txnrd2*). SAV1 KD cells showed antioxidant defenses by upregulating mRNA levels of *Gpx1, Cat, Sod2, Prdx5, Txn2,*and *Txnrd2* (Fig. 4E)^25^.

### Knockdown of SAV1 regulates metabolic signatures and signaling pathways in C2C12 myoblasts

**Figure 5:**
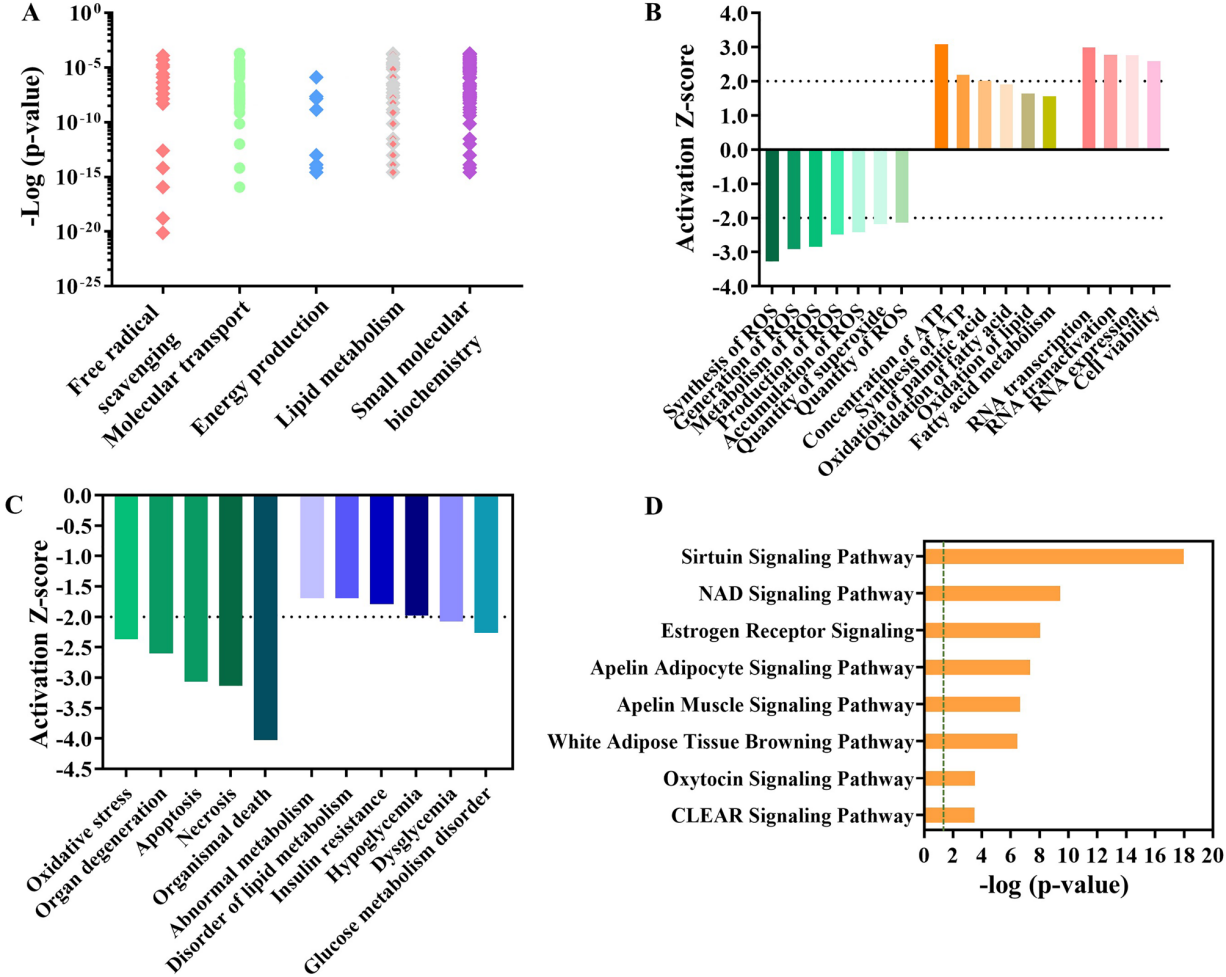
Ingenuity pathway analysis of the mRNA data. (**A**) The comparison of mRNA expression pattern in SAV1 KD cells to the ingenuity knowledge database revealed the top significantly upregulated molecular and cellular signatures in SAV1 KD myoblasts. Right-tailed Fisher’s exact test; p < 0.05 was considered statistically significant. (**B**) The over-represented biological functions in SAV1 KD cells included reduced reactive oxygen species (ROS) synthesis and increased energy production, RNA transcription, and cell viability. The Z score was used to calculate the data interpretation. Z-score ≥ 2.0 (activation) and Z-score ≤ -2.0 (inhibition). The gray dash line indicates the threshold of significance. (**C**) SAV1 KD was predicted to downregulate organism death, oxidative stress, and metabolic disorders. Z-score ≥ 2.0 (activation) and Z-score ≤ -2.0 (inhibition). (**D**) The top upregulated canonical pathways in SAV1 KD cells. Right-tailed Fisher’s exact test; green dash line indicating the threshold p value (p = 0.05).

To gain insight into how differential gene expression may affect cell processes, biological functions, and diseases, we applied the ingenuity pathway analysis (IPA) network generation algorithm to compare expression patterns of molecules in our RT-qPCR data to what is known in the ingenuity knowledge base^26^. The top significantly upregulated molecular and cellular functions, as the result of SAV1 KD, included free radical scavenging, molecular transport, energy production, lipid metabolism, and small molecular biochemistry (P value ranging from 1.32 × 10^–4^ to 1.20 × 10^–16^, Fig. 5A). By IPA analysis, the active free radical scavenging and molecular transport functions are associated with reducing the synthesis, generation, metabolism, and accumulation of ROS. The energy production, lipid metabolism, and small molecular biochemistry functions involve higher cellular ATP concentrations. SAV1 KD activated gene expression (i.e., RNA transcription and transactivation) and increased cell viability (Fig. 5B). In addition, SAV1 KD is postulated to inhibit oxidative stress, organismal death, and apoptosis, and to reduce glucose metabolism disorders (Fig. 5C). The pathway analysis identified the top upregulated signaling pathways including sirtuin signaling, NAD (nicotinamide adenine dinucleotide) signaling, and apelin signaling pathways (Fig. 5D).

**Figure 6:**
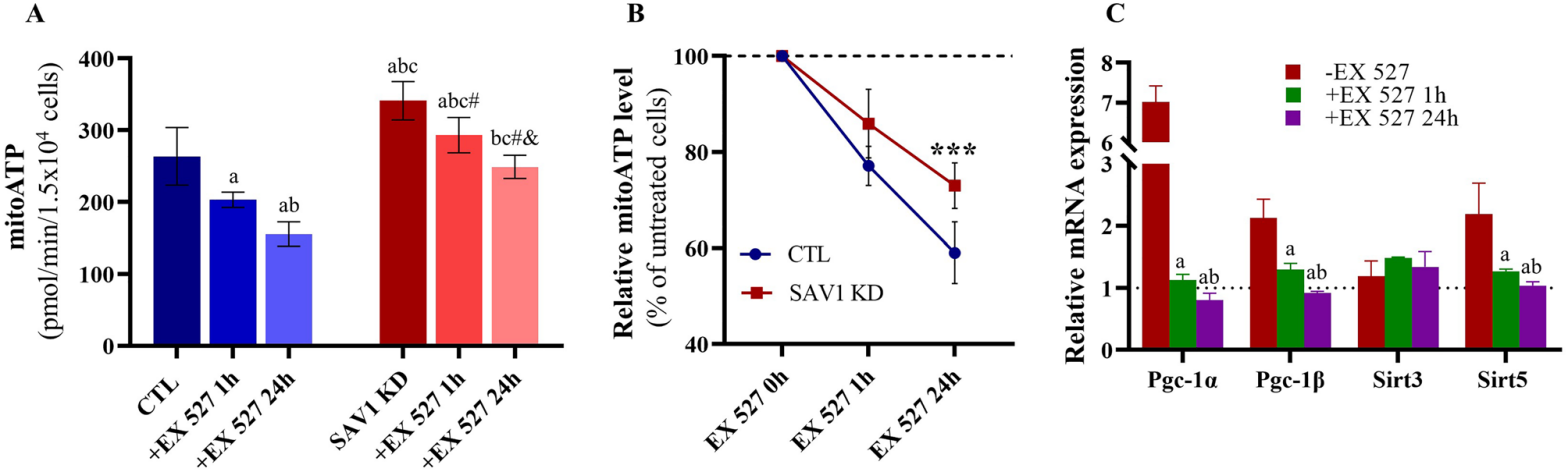
Effects of sirtuin 1 (Sirt1) inhibition on mitochondrial ATP production. (**A**) Representative analysis of mitochondrial ATP generation of SAV1 KD and CTL cells in the absence and presence of the Sirt1 inhibitor EX 527 for 1 h and 24 h under basal conditions. One-way ANOVA with Tukey’s multiple comparisons test; p ≤ 0.05 is statistically significant. Data are mean ± SD, n = 3 independent measurements. a = significant vs CTL without EX 527; b = significant vs CTL + EX 527 1 h; c = significant vs CTL + EX 527 24 h; # = significant vs SAV1KD without EX 527; and & = significant vs SAV1 KD + EX 527 1 h. (**B**) SAV1 KD cells are more resistant to EX 527-induced reduction of mitochondrial ATP production. Unpaired t test for comparison between SAV1 KD and CTL cells within the same time period. ***p < 0.001. (**C**) Sirt1 inhibition induced a time-dependent suppression of mRNA levels of *Pgc-1α*, *Pgc-1β*, and mitochondrial *Sirt5*. One-way ANOVA with Tukey’s multiple comparisons test; p ≤ 0.05 is statistically significant. Data are mean ± SD, n = 3 independent measurements. a = significance vs. SAV1 KD cells in the absence of EX 527 within the same gene; b = significance vs. SAV1 KD cells treated with EX 527 1 h within the same gene.

Because sirtuin signaling is the most highly enriched pathway after SAV1 KD, we examined the effect of Sirt1 on mitochondrial ATP production. CTL and SAV1 KD cells were pretreated with the Sirt1 inhibitor EX 527 (50 μm) for 1 h or for up to 24 h, and we measured ATP produced by mitochondrial OXPHOS (Fig. 6A). In the presence of EX 527, cells exhibited a time-dependent reduction in mitochondrial ATP production; this trend was more pronounced in CTL than in SAV1 KD cells. After 1 h of EX 527 treatment, we noted a 23% reduction in mitoATP in CTL cells and a 14% reduction in SAV1 KD cells as compared to their untreated counterparts. Continuous exposure to EX 527 for 24 h resulted in a 41% reduction in mitoATP in CTL cells and a 27% reduction of mitoATP in SAV1 KD cells (*P* < 0.001, Fig. 6B). These findings suggest Sirt1 facilitated OXPHOS-coupled ATP production and confirmed higher levels of Sirt1 expression in SAV1 KD cells. In addition, Sirt1 inhibition altered the relative mRNA expressions of *Pgc-1α*, *Pgc-1β,* and *Sirt5* in SAV1 KD cells as compared to untreated counterparts (SAV1 KD cells in the absence of EX 527; Fig. 6C). The expressions of *Pgc-1α*, *Pgc-1β*, *and Sirt5* mRNA were significantly suppressed after 1 h of EX 527 treatment and were further reduced after 24 h of EX 527 treatment. In contrast, Sirt1 inhibition did not affect mRNA level of *Sirt3*.

### Knockdown of SAV1 enhances metabolic adaptability to environmental change

**Figure 7:**
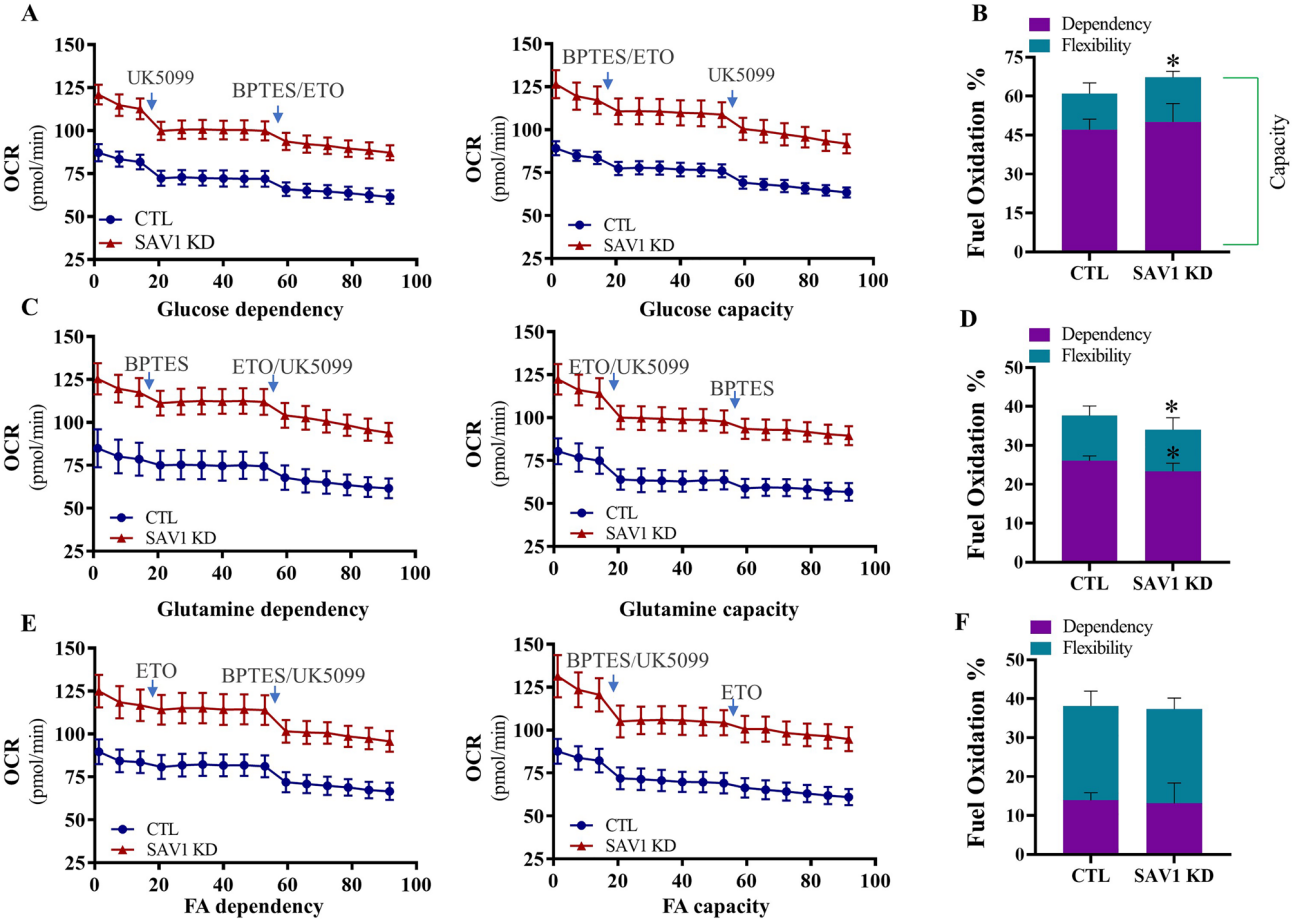
Distinct effects of SAV1 KD cells on mitochondrial flexibility for using fuel. (**A**) Representative traces of measurements of oxygen consumption rate (OCR) showing the dependency on and capacity for glucose oxidation after injection of inhibitors (individually or in combination) as indicated in the graph. UK5099: inhibits glucose oxidation; etomoxir (ETO): inhibits fatty acid oxidation (FAO); BPTES: inhibits glutamine oxidations. (**B**) SAV1 KD cells had significantly enhanced mitochondrial capacity to use glucose oxidation to compensate for inhibition of glutamine oxidation and FAO. Flexibility is the difference between capacity and dependency. *p < 0.05. (**C**) Representative traces of OCR measurements showing the dependency on and capacity for glutamine oxidation after injection of inhibitors (individually or in combination) as indicated in the graph. (**D**) SAV1 KD cells had a significantly lower dependency on and capacity in glutamine oxidation than CTL cells to compensate for the inhibition of glucose oxidation and FAO. *p < 0.05. (**E**) Representative traces of OCR measurements showing the dependency on and capacity for FAO after injection of inhibitors (individually or in combination) as indicated in the graph. (**F**) SAV1 KD and CTL cells had similar flexibility in FAO when both glucose and glutamine oxidation pathways were blocked. Unpaired t test; Data are mean ± SD, n = 4 independent measurements.

Mitochondrial ATP production is fueled by 3 substrate oxidation pathways: glucose-coupled OXPHOS, fatty acid oxidation (FAO)-coupled OXPHOS, and glutamine-coupled OXPHOS^27,28^. Using pharmacological intervention, we examined mitochondrial fuel dependency and capacity to assess the effect of SAV1 KD on mitochondrial fuel flexibility in oxidizing each fuel source. Flexibility (%) is determined by the difference between fuel capacity (%) and dependency (%). Mitochondrial OCR was measured in real time after sequential injections of fuel pathway inhibitors alone or in combination. The inhibitors UK5099 (targets glucose oxidation), BPTES (targets glutamine oxidation), and etomoxir (ETO; targets FAO) were given to cells as shown in Fig. 7. The fuel preferences for both CTL and SAV1 KD cells were glucose > glutamine > fatty acid. No significant difference was detected in the dependency of glucose as a fuel between SAV1 KD and CTL cells (50.07% ± 7.08% vs 47.11% ± 4.05%, respectively). When glutamine and FAO pathways were inhibited by a BPTES/ETO mixture, SAV1 KD significantly increased the cells’ ability to conduct glucose oxidation (67.22% ± 2.37% vs 60.99% ± 4.10%; SAV1 KD vs CTL cells). As a result, mitochondrial flexibility was 13.88% in CTL and 17.15% in SAV1 KD cells (Fig. 7A,B). This suggests that flexible metabolism in SAV1 KD cells allowed them to use glucose when alternative fuels (glutamine and fatty acid) were not available. In addition, SAV1 KD cells showed a significantly reduced dependency on glutamine as a fuel source (23.34% ± 2.09% vs 26.09% ± 1.23%; SAV1 KD vs CTL cells) and a significantly reduced capacity to oxidize glutamine (34.06% ± 3.04% vs 37.66% ± 2.47%) when glucose and FAO were inhibited simultaneously by UK5099/ETO. However, we found no marked differences in flexibility of glutamine oxidation between CTL and SAV1 KD cells (Fig. 7C,D). Finally, we measured cell flexibility in oxidizing endogenous fatty acid. No difference was detected in the dependency of fatty acid as a fuel (13.14% ± 5.24% vs 13.91% ± 1.94%; SAV1 KD vs CTL cells). When glucose/glutamine oxidation was inhibited by UK5099/BPTES, both CTL and SAV1 KD cells exhibited a similar capacity to conduct FAO (37.34% ± 2.85% vs 38.14% ± 3.82%; SAV1 KD vs CTL cells, Fig. 7E,F). In short, our data suggest glucose serves as a major substrate to meet cell energy requirements. SAV1 KD enhanced the cells’ capacity to utilize glucose oxidation in replacing other fuel substrates when scarce, indicating the critical role of glucose oxidation for ATP production.

**Figure 8:**
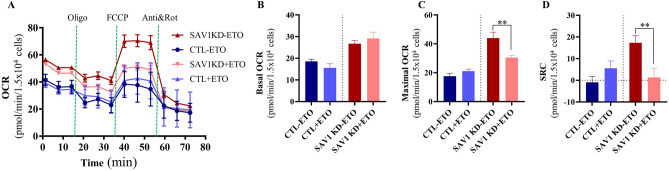
SAV1 KD cells had enhanced ability to oxidize endogenous fatty acid under limited glucose with bovine serum albumin. (**A**) Representative traces of real-time OCR measurements in both SAV1 KD and CTL cells showing the basal respiration and OCR changes in response to sequential injections of inhibitors for mitochondrial oxidative phosphorylation. (**B**) Under lower substrate demand, basal OCR was similar in CTL cells with or without ETO treatment. Similarly, no difference was seen in basal OCR in SAV1 KD cells with or without ETO treatment. (**C**) After being bioenergetically stressed with FCCP to increase substrate demand, SAV1 KD cells had a substantially enhanced capacity to use FAO as compared to CTL cells. There was a significant difference in maximal OCR in SAV1 KD cells between conditions with and without ETO treatment, suggesting that endogenous FA was being oxidized. (**D**) SAV1 KD cells without ETO treatment showed a significant enhancement in spare respiratory capacity (SRC) over those with ETO treatment. Unpaired t test between the same cell type in the presence and absence of ETO. Data are mean ± SD, n = 3 independent measurements. **p < 0.01.

We combined ETO pretreatment with mitochondrial stress testing to compare the abilities of CTL and SAV1 KD myoblasts to oxidize endogenous fatty acids in an environment with limited glucose and no glutamine (Fig. 8A). Basal and maximal respiration were measured in a substrate-reduced growth medium supplemented with 2 mM glucose, 0.5 mM L-carnitine, and bovine serum albumin. ETO inhibits carnitine palmitoyl transferase 1A to block the transfer of fatty acid from the cytosol to the mitochondria. ETO pretreatment was used to probe for the utilization of endogenous fatty acid and to determine the contribution of fatty acid to basal and maximal OCR. The basal OCR of CTL cells without ETO (CTL-ETO, 18.58 ± 0.93 pmol O_2_/min) was similar to that of CTL cells treated with ETO (CTL + ETO, 15.53 ± 1.94 pmol O_2_/min, Fig. 8B). Similarly, the basal OCR of SAV1KD cells without ETO (SAV1 KD-ETO, 26.74 ± 1.39 pmol/min) was similar to that in ETO-treated cells (SAV1 KD + ETO, 29.20 ± 2.89 pmol/min). When cells were treated with FCCP to introduce bioenergetic stress, the average maximal OCR did not increase in the CTL-ETO group as compared to the CTL + ETO group (17.64 ± 1.93 vs 21.05 ± 1.46 pmol/min, Fig. 8C). Interestingly, a noteworthy increase in maximal OCR was seen in the SAV1 KD-ETO group as compared to the SAV1 KD + ETO group (44.05 ± 4.07 vs 30.47 ± 1.39 pmol/min, Fig. 8C). Although the difference in SRC in CTL groups with and without ETO treatment was subtle, we found a significant reduction in the SAV1 KD group with ETO as compared to that without ETO (Fig. 8D); these results imply that SAV1 KD in myoblasts significantly improved maximal capacity to use endogenous FAO to support the ATP demand and SAV1 KD cells gained mitochondrial reserve when energetic stress was induced and alternative energy substrates were limited.

## Discussion

Regulating cell proliferation and survival in response to environmental fluctuations is an energy-demanding process. In this study, we found that Hippo pathway inhibition via SAV1 KD in C2C12 myoblasts had numerous substantial effects on metabolism, resulting in improved metabolic flexibility in response to a flux in fuel substrates. These effects included enhanced mitochondrial biogenesis and glycolytic capacity, transcriptional activation of genes directing metabolic reprogramming, ROS scavenging, and, most importantly, metabolic flexibility. Compared with control cells, SAV1 KD cells had a higher SRC and more glycolytic reserves to cope with increased energy demands under metabolic stress. Furthermore, SAV1 KD cells had an increased capacity and flexibility for oxidizing glucose when glutamine and endogenous FAO were blocked. In addition to regulating glucose metabolism, SAV1 KD promoted endogenous FAO when the glucose supply was limited, reflecting a high metabolic plasticity of SAV1 KD cells for fueling mitochondrial respiration. Finally, we found that upregulation of the sirtuin pathway contributed to enhanced mitochondrial ATP production in SAV1 KD cells.

Using the extracellular flux analysis to measure real-time metabolism, we found that SAV1 KD regulated cell dependence on mitochondrial OXPHOS and glycolysis according to the particular environment. Under normal conditions with sufficient glucose (10 mM) and pyruvate (1 mM) levels, SAV1 KD myoblasts performed better than CTL cells in bioenergetic metrics such as basal, ATP-linked, and maximal OCR as well as non-mitochondrial OCR; these findings reflect a higher overall energy state and more active cell oxidative reactions. The increased mitochondrial SRC and ATP-linked OCR in SAV1 KD cells contributed to a significantly higher bioenergetic health index, which provides additional energy reserves that enable cells to overcome stress. Moreover, after cells were starved of glucose and pyruvate for 1 h, when glucose was injected into the environment, SAV1 KD cells showed a remarkably higher ability to take up glucose for glycolysis than did CTL cells. This augmented ability may be caused by accelerated enzymatic reactions in the glycolysis pathway that speed up ATP generation. In fact, we detected mRNA overexpression of 3 key enzymes—hexokinase (*Hk2*), pyruvate kinase (*Pkm*), and phosphofructokinase (*Pfkm*)—that catalyze glucose to pyruvate in SAV1 KD cells. Furthermore, when mitochondrial ATP synthase was blocked by oligomycin to terminate OXPHOS, the ECAR was slightly reduced in CTL cells, indicating they lack either the potential to increase ATP production from glycolysis or the glycolytic reserve to meet energy-demanding situations. In contrast, SAV1 KD cells exhibited a higher glycolytic capacity to maximize glucose breakdown to produce more ATP via glycolysis. These efficient shifts in metabolism to adapt to environmental changes give myoblasts a survival advantage under environmental challenges.

Skeletal muscle has a high metabolic activity level and requires a rapid increase in the rate of ATP production during periods of increased metabolic demands such as physical activities^29^. Glucose serves as a main energy substrate because glucose metabolism is the most efficient way to provide ATP in skeletal muscle. Aside from improved bioenergetic reserves, SAV1 KD further increased glucose oxidation when oxidation pathways for alternative fuel sources (glutamine and fatty acids) were interrupted. Interestingly, our results show a different scenario for how SAV1 KD cells used endogenous fatty acids to respond to a stress energy demand. When glucose was reduced from the saturating concentration (10 mM) to a limited amount (2 mM), SAV1 KD cells were able to increase their mitochondrial capacity to oxidize endogenous fatty acids. Under conditions of higher bioenergy demand (injection of FCCP), SAV1 KD cells (SAV1 KD-ETO group in Fig. 8) had a maximal respiration rate that was 250% higher than that in CTL cells (CTL-ETO group). SAV1 KD cells showed a much larger difference than CTL cells in the FCCP-stimulated maximal OCR between conditions without and with ETO, which suggests that enhanced OXPHOS by SAV1 KD cells is derived exclusively from oxidizing fatty acid. However, SAV1 KD cells lost the potency to enhance fatty acid oxidative capacity when the glucose oxidation pathway was completely inhibited. In the presence of UK5099, an inhibitor of mitochondrial pyruvate carrier, no difference was seen in FAO capacity between SAV1 KD and CTL cells (Fig. 7F). This implicit FAO signature in SAV1 KD cells is largely influenced by glucose oxidation, which suggests a complex dynamic relationship between FAO and glucose oxidation. Of importance, the enhanced ability to oxidize fatty acids under low glucose conditions provides an alternative way to supply bioenergy needs in myoblasts and may confer benefits by enhancing skeletal muscle mass and function. Indeed, an attenuated myogenic capacity to transition between glucose and fatty acid use for energy production is a key factor in the pathogenesis of metabolic diseases characterized by a loss of metabolic flexibility^30^. Our study showed a complete inhibition of glucose oxidation in SAV1 KD cells diminished the enhancement of FAO, indicating SAV1 KD cannot simply enhance the substrate replacement from glucose to fatty acid in myoblasts. This finding suggests a subtle balance may exist between glucose and fatty acid utilization for maintaining metabolic flexibility.

In mammals, the sirtuin family contains 7 members of class III histone deacetylases (*Sirt 1-Sirt7*) with different cell localizations^31,32^. They participate in several biological processes such as metabolism, cell survival, and redox homeostasis. Here, we found that sirtuin signaling was the top upregulated pathway involved in mitochondrial biogenesis after SAV1 KD. When treated with the Sirt1 inhibitor EX 527, SAV1 KD cells showed less reduction in OXPHOS-dependent ATP production than did CTL cells. In analyzing the findings in the time-course study, we found that the differential response to EX 527 between CTL and SAV1 KD groups became more apparent after 24 h of exposure. This confirms the prolonged effect of Sirt1 on energy oxidative capacity. Of note, mitoATP production in SAV1 KD cells did not return to the levels of CTL cells after Sirt1 inhibition, suggesting that genes or sirtuin family members other than Sirt1 may independently contribute to higher mitoATP synthesis. Previous studies have suggested that mitochondrial sirtuins Sirt3 and Sirt5 modulate cellular metabolism^33,34^. Higher content of mitochondrial Sirt3 is compatible with ATP production, and the loss of Sirt3 reduces intracellular ATP levels. Sirt3 knockout mice showed impaired oxygen consumption in parallel with increased oxidative stress in skeletal muscle. Moreover, Sirt3 KD in C2C12 myoblasts reduced basal and maximal OCR^35^. Sirt5, like Sirt3, regulates the efficiency of OXPHOS and the catabolism of glucose and FAO^33^. Supporting the results of the above studies, we found that *Sirt3* and *Sirt5* mRNA levels increased in parallel with *Sirt1* in SAV1 KD cells as compared to CTL cells. However, the Sirt1 inhibitor EX 527 had different individual effects on *Sirt 3* and *Sirt5* mRNA levels; *Sirt5* was downregulated, and *Sirt* 3 expression was not affected. Because of the lack of specific pharmacological agents, we did not target Sirt3 and Sirt5 individually, in combination, or in different combinations with Sirt1, to examine individual or combined effects on mitochondrial metabolism. In addition to affecting mitochondrial sirtuins, we found that inhibition of Sirt1 in SAV1 KD myoblasts abolished the increased expression of the well-characterized metabolic master regulator of *PGC-1α,* supporting the supposition that Sirt 1-mediated regulation of *PGC-1α* activity may play a role in mitochondrial respiration and the utilization of substrates in adapting energy metabolism as needed.

The ability to improve metabolic plasticity by affecting SRC, glycolytic reserves, glucose oxidation, and FAO such as what we found in SAV1 KD cells may have clinical potential in treating metabolic dysfunction as patients with type 2 diabetes mellitus and obesity often have impaired skeletal muscle metabolic homoeostasis^36^. Blunted mitochondrial function and lower ATP production in skeletal muscle of obese and insulin-resistant individuals have been found to contribute to the defect in glucose and fatty acid oxidative metabolism seen in these conditions, which is accompanied by increased oxidative stress^37^. Moreover, dysregulation of well-characterized genes involved in mitochondrial biogenesis such as *Sirt1, PGC-1α,* and *Sirt3* is implicated in the pathogenesis of diabetes^38^. Sirt1 activates PGC-1α by deacetylation, which leads to the upregulation of PGC-1α-induced genes for mitochondrial bioenergetics and FAO^39,40^. Here, we demonstrate the beneficial effects of SAV1 KD in myoblasts, including increased expression of *Sirt1*, *PGC-1α*, and *PGC-1β* enhanced mitochondrial SRC; and reduced oxidative stress. According to our data, Sirt1 acts as an upstream activator of *PGC-1α* and *PGC-1β* expression. Knowledge-based causal analysis prediction algorithms predict SAV1 KD in myoblasts may reduce a variety of abnormal metabolism, ranging from glucose and lipid metabolism disorders to insulin resistance. Similar to the positive regulation of mitochondrial biogenesis, by measuring the real-time ECAR, we found that SAV1 KD promoted glycolytic capacity in response to mitochondrial ATP inhibition, adding another layer of metabolic adaptability. This indicates SAV1 KD cells are capable of redirecting energy reliance from OXPHOS toward glycolysis to compensate for decreased mitochondrial ATP production. In contrast, CTL cells showed no glycolytic reserve in the glycolysis stress assay.

In conclusion, we present clear evidence of the positive role of Hippo pathway inhibition via SAV1 KD on regulating mitochondrial function and metabolic flexibility to environmental changes. We are aware of our study limitations as cell bioenergetics measured by the extracellular flux assays may be influenced by cell density, passages, and viability and by experimental conditions. Nevertheless, our measurements provide real-time, noninvasive assessments of cell metabolic fluctuations. In the future, we will use the extracellular flux analysis to examine skeletal muscle metabolism on myogenic-specific SAV1 knockout and will detail molecular and signaling pathways that drive metabolic flexibility.

## Methods

### SAV1 knockdown in C2C12 myoblasts by siRNA transfection

Mouse C2C12 myoblasts (ATCC, Manassas, VA, USA) were cultured in 6-well tissue culture plates with high glucose Dulbecco's Modified Eagle's medium (DMEM) containing 10% fetal bovine serum (FBS) and 1% penicillin–streptomycin (PS) in a 5% CO_2_ incubator at 37 °C. Silencer select SAV1 siRNA (assay ID 182232, Thermo Fisher Scientific, Waltham, MA, USA)-Lipofectamine RNAiMAX complexes or silencer negative control siRNA (#AM4611)-Lipofectamine RNAiMAX complexes were transfected into myoblasts according to the manufacturer's procedures (Thermo Fisher Scientific) for 24–72 h. C2C12 myoblasts transfected with negative control siRNA were referred to as CTL, and those transfected with SAV1 siRNA were referred to as SAV1 KD. Before using transfected cells in the metabolic and molecular analyses, trypan blue exclusion assays were conducted to quantify viable cells under a light microscope (Olympus CKX31) as previously described^41,42^. No live animals were used in the study.

### Quantitative real-time RT-PCR

At 24, 48, and 72 h after siRNA transfection, myoblasts were washed with Dulbecco's phosphate-buffered saline (DPBS) and collected for RNA isolation (RNase Plus Micro Kit, Qiagen, Hilden, Germany), respectively. Total RNA (2 μg) was reverse transcribed using high-capacity RNA-to-cDNA kit (Invitrogen, Waltham, MA, USA) and T100 thermal cycler (Bio-Rad, Hercules, CA, USA). qPCR was performed using the TaqMan Gene Expression Master Mix (Invitrogen) and QuantStudio 6 Real-Time PCR System (Life Technologies, Grand Island, NY, USA). SAV1-specific primers/probes (assay ID Mm01292174_m1), 18S rRNA endogenous control (VIC/MGB Probe), and other primers/probes used in the current study were purchased from Thermo Fisher Scientific. The relative expression of RNA was calculated using QuantStudio Real Time PCR software (the ΔΔCt method). All experiments were performed in triplicate in three independent experiments. To examine the effect of Sirt1 inhibitor EX 527 on the expressions of *Pgc-1α*, *Pgc-1β, Sirt3,* and *Sirt5* mRNA in CTL and SAV1 KD cells, we pretreated siRNA transfected cells with 50 μm EX 527 for 1 h or up to 24 h before cell harvesting for RNA isolation.

### Western blot analysis

At 72 h after siRNA transfection, cells were washed with DPBS and lysed in an ice-cold lysis buffer containing a protease inhibitor cocktail (Roche, Basal, Switzerland) on ice. Protein isolation, antibody used and western blot procedures were performed as described previously^19^. Active anti-YAP1 (ab205270) was purchased from abcam, and anti-β-actin (#4970) was purchased from Cell Signaling Technology. Semi-quantitative analysis of protein bands was performed by using ImageJ (https://imagej.nih.gov/ij/, ImageJ bundled with 64-bit Java 8).

### Real-time cell metabolic analysis and bioenergetics measurements

All metabolic assays were conducted according to manufacturer’s protocols, unless stated otherwise (Agilent, Santa Clara, CA, USA). Assay kits, chemical compounds, media and XF96 PDL microplates were purchased from Agilent. Cell culture microplates were pre-coated with 2% gelatin solution (Millipore Sigma, St. Louis, MO, USA) at room temperature for 4 h and dried overnight. C2C12 myoblasts transfected with either negative control siRNA (CTL) or SAV1 siRNA (SAV1 KD) for 48 h were seeded in XF96 cell culture microplates (1.5 × 10^4^ cells/well) in DMEM containing 10% FBS and 1% PS and incubated for 24 h in a 37 °C incubator containing 5% CO_2_. The Seahorse XFe96 Extracellular Flux Analyzer was used to measure OCR and ECAR of live cells. Each metabolic rate data summary was normalized to represent equal numbers of cells (1.5 × 10^4^ cells) in both CTL and SAV1 KD groups.

#### ATP rate assay

On the day of the assay, cells were washed once and equilibrated with XF DMEM assay medium (pH 7.4) supplemented with 10 mM glucose, 1 mM pyruvate, and 2 mM glutamine in a 37 °C, non-CO_2_ incubator for 1 h. After determining basal OCR and ECAR in parallel, we sequentially injected oligomycin (final concentration, 1.5 μM) and a mix of the mitochondrial electron transport chain inhibitors rotenone and antimycin A (Rot&AA, 0.5 µM, XF Real-Time ATP Rate Assay kit) into cells and made simultaneous OCR/ECAR measurements. Immediately after completing all measurements, we calculated cell numbers per well and used those numbers to normalize the OCR/ECAR values. Assays were independently performed 4 times. The conversion of ATP-coupled OCR into the mitochondrial ATP production rate, the calculation of the proton efflux rate from ECAR plus the buffer factor of the assay medium, and the glycolytic ATP production rate were completed by on-line seahorse analytics as provided by the manufacturer. To quantify mitochondrial ATP production change in response to EX 527 in CTL and SAV1 KD cells, we pretreated cells with 50 μm EX 527 for 1 h or up to 24 h before the start of the ATP rate assays at 72 h post-transfection. We performed 3 independent measurements. Individual measurements were generated by using 10–14 technique replications per cell group.

#### Mitochondrial stress tests

Cells were cultured and seeded in XF96 culture microplates as described above. Before performing the stress assay, we equilibrated the cells with XF DMEM assay medium (pH7.4) supplemented with 10 mM glucose, 1 mM pyruvate, and 2 mM glutamine in a 37 °C, non-CO_2_ incubator for 1 h. After concurrently determining basal OCR and ECAR, we sequentially injected oligomycin (final concentration: 1.5 μM), FCCP (1.0 μM), and the mito-inhibitors Rot&AA (0.5 µM, XF Cell Mito Stress Test kit) into the cell cultures and made simultaneous OCR/ECAR measurements. Individual measurements were produced from 28 technique replications per cell group. We performed 3 independent measurements. The data were generated by wave 2.6.1; key parameter equations were described by the report generator user guide and analyzed by on-line seahorse analytics provided by the manufacturer. The bioenergetic health index was calculated using a formula described previously^43^.

#### Glycolysis stress tests

On assay day, cells were equilibrated with XF DMEM base medium (pH, 7.4) supplemented with 2 mM glutamine in a 37 °C, non-CO_2_ incubator for 1 h before measuring baseline ECAR by using the Seahorse XFe96 Extracellular Flux Analyzer. After determining basal ECAR, we sequentially injected glucose (final concentration, 10 mM), oligomycin (1.0 μM), and 2-DG (50 mM, XF Glycolysis Stress Test kit) into the cell cultures and made simultaneous ECAR/OCR measurements. Individual measurements were produced from 39 technique replications per cell group. We performed 3 independent measurements. Glycolytic function key parameter equations were described by the manufacturer.

#### Endogenous fatty acid oxidation stress tests

After seeding cells for 4 h in XF96 cell culture microplates (1.5 × 10^4^ cells/well) in DMEM medium containing 10% FBS and 1% PS, we switched the culture medium to a substrate-limited growth DMEM medium supplemented with 0.5 mM glucose, 1 mM glutamine, 1% FBS, and 0.5 mM L-carnitine overnight in a 37 °C, 5% CO_2_ incubator. On the assay day, cells were washed once and equilibrated with XF DMEM substrate-limiting medium (pH 7.4) supplemented with 2.0 mM glucose and 0.5 mM L-carnitine in a 37 °C, non-CO_2_ incubator for 1 h. We then added ETO (final concentration: 40 μM) to the appropriate wells and incubated cells in a 37 °C, non-CO_2_ incubator for an additional 15 min. Next, we added 30uL of BSA (XF Palmitate-BSA FAO Substrate Kit) to each well before measuring baseline OCR by using the Seahorse XFe96 Extracellular Flux Analyzer. After determining basal OCR, we sequentially injected oligomycin (1.5 μM), FCCP (2.0 μM), and mito-inhibitors Rot&AA (0.5 µM, XF Cell Mito Stress Test kit) into cells and made the OCR measurements. Individual measurements were produced from 10–11 technique replications per cell group. We performed 3 independent measurements. The data were analyzed using analytics algorithms provided by the manufacturer.

#### Mitochondrial fuel flexibility tests

We equilibrated cells with XF DMEM assay medium (pH 7.4) supplemented with 10 mM glucose, 1 mM pyruvate, and 2 mM glutamine in a 37 °C, non-CO_2_ incubator for 1 h before measuring baseline OCR. BPTES (final concentration, 3.0 μM), ETO (4.0 μM), and UK099 (2.0 μM), either individually or in combination, were serially added to cells, and OCR values were taken (XF Mito Fuel Flex Assay Kit). The percentage of mitochondrial dependency, the capacity and flexibility on glucose, glutamine, or FAO, was calculated according to the formula and software provided by the manufacturer. Individual measurements were produced from 5–8 technique replications per cell group. We performed 4 independent measurements.

### Cell proliferation with EdU labeling and cell viability under oxidative stress

To label proliferating cells, we added EdU (final concentration, 10 μM, Thermo Fisher Scientific) to culture media at 70 h after siRNA transfection. After 2 h (72 h after siRNA transfection), the culture supernatant was aspirated, and the cells were washed 2 times with PBS before being fixed with 4% paraformaldehyde. EdU staining of cells and quantification were performed as previously described^19^ (Click-iT EdU cell proliferation kit for imaging, Alexa Fluor 555 dye, Thermo Fisher Scientific).

To evaluate if SAV1 KD affects the anti-oxidative effects of C2C12 myoblasts, at 72 h after siRNA transfection, CTL and SAV1 KD cells (2.5 × 10^4^ cells/well in a 96-well plate) were treated with DMEM (with no phenol red) containing 100–400 μM H_2_O_2_ for 2 h in parallel. Then we measured cell viability by using MTT (3-(4,5-dimethylthiazol 2-yl)-2,5-diphenyltetrazolium bromide) according to the manufacturer’s instruction (Vybrant MTT Cell Proliferation Assay kit, Molecular Probes, Thermo Fisher). The absorbance at 570 nm was measured by a microplate reader (TECAN).

### Mitochondrial mass and analysis of mitochondrial membrane potential

We determined mitochondrial mass by measuring the mtDNA/nDNA ratio as described previously^44^. Briefly, 48-h post siRNA transfection, CTL and SAV1 KD cells were washed with cold DPBS. DNA was isolated (QIAamp DNA Mini Kit, Qiagen) and quantified (NanoDrop). Real-time quantitative PCR was conducted according to the manufacturer’s instruction (Power SYBR Green PCR Master Mix, Thermo Fisher Scientific). Genes for the mitochondrially encoded NADH:ubiquinone oxidoreductase core subunit 1 (*Mt-Nd1*) and nuclear hexokinase 2 (*Hk2*) were used to quantify the mtDNA/nDNA ratio^44^.

At 48 h after siRNA transfection, CTL and SAV1 KD cells (2 × 10^5^ cells/ sample) were harvested by trypsinization and then centrifuged. Cell pellets were resuspended in 1 ml of warm PBS. The positive staining controls comprised one CTL and SAV1 KD cell sample pretreated with CCCP (final concentration, 50 μM) in a 5% CO_2_ incubator at 37 °C for 5 min. JC-1 dye (final concentration of 2 μM, MitoProbe JC-1 assay kit, ThermoFisher) was added to each cell sample, and cells were incubated in a 5% CO_2_ incubator at 37 °C for 20 min. Cells were washed with warm PBS and centrifuged. We removed the supernatant and resuspended the cell pellets in 300 μl PBS. Cell samples were immediately counted in a BD flow cytometer (LSRII) according to the protocol provided by the manufacturer. The ratio of red and green fluorescence intensities was determined by flowJo_v10.8.1 software. The MMP of each cell sample was calculated by the ratio of red fluorescence intensity divided by that of green fluorescence intensity. Four independent experiments were performed.

### Bioinformatic analysis

qRT-PCR data were uploaded into the online IPA network algorithm (https://analysis.ingenuity.com) to compare expression patterns with the large-scale gene expression network from the ingenuity knowledge base. We performed core analyses such as identifying regulators and gene network interactions and predicting enriched pathways, diseases, and function.

### Statistical analysis

Data were expressed as the mean ± standard deviation unless otherwise stated. The unpaired t test was used to determine statistical significance between two groups. For more than 2 groups, we used a one-way analysis of variance with Tukey’s multiple comparisons test (Graph Pad Prism 9.3.1). *P* < 0.05 was considered statistically significant. For IPA data interpretation, a P value of overlap was calculated by using the right-tailed Fisher’s exact test, and *P* < 0.05 was considered statistically significant; the z score was used to calculate activation or inhibition of predicted molecular functions and diseases: z-score ≥ 2.0 (activation) and ≤ -2.0 (inhibition).

## Supplementary Information

Supplementary Information.

## Data Availability

The data that support the findings of this study are available from the corresponding author upon reasonable request.

## References

[1] Bonora M (2012). ATP synthesis and storage. Purinergic Signal.

[2] Martínez-Reyes I, Chandel NS (2020). Mitochondrial TCA cycle metabolites control physiology and disease. Nat. Commun..

[3] Webster KA (2003). Evolution of the coordinate regulation of glycolytic enzyme genes by hypoxia. J. Exp. Biol..

[4] Vander Heiden MG, Cantley LC, Thompson CB (2009). Understanding the Warburg effect: the metabolic requirements of cell proliferation. Science.

[5] Vander Heiden MG (2010). Evidence for an alternative glycolytic pathway in rapidly proliferating cells. Science.

[6] Lunt SY, Vander Heiden MG (2011). Aerobic glycolysis: Meeting the metabolic requirements of cell proliferation. Annu. Rev. Cell Dev. Biol..

[7] Oyewole AO, Birch-Machin MA (2015). Mitochondria-targeted antioxidants. Faseb J..

[8] Schieber M, Chandel NS (2014). ROS function in redox signaling and oxidative stress. Curr. Biol..

[9] Snezhkina AV (2019). ROS generation and antioxidant defense systems in normal and malignant cells. Oxid. Med. Cell Longev..

[10] Desler C (2012). Is there a link between mitochondrial reserve respiratory capacity and aging?. J. Aging Res..

[11] Schwer B, Verdin E (2008). Conserved metabolic regulatory functions of sirtuins. Cell Metab..

[12] Cantó C, Auwerx J (2009). PGC-1alpha, SIRT1 and AMPK, an energy sensing network that controls energy expenditure. Curr. Opin. Lipidol..

[13] Mittler R (2002). Oxidative stress, antioxidants and stress tolerance. Trends Plant Sci..

[14] Smith RL, Soeters MR, Wüst RCI, Houtkooper RH (2018). Metabolic flexibility as an adaptation to energy resources and requirements in health and disease. Endocr. Rev..

[15] Dumont NA, Bentzinger CF, Sincennes MC, Rudnicki MA (2015). Satellite cells and skeletal muscle regeneration. Compr. Physiol..

[16] Relaix F, Marcelle C (2009). Muscle stem cells. Curr. Opin. Cell. Biol..

[17] Ramos A, Camargo FD (2012). The Hippo signaling pathway and stem cell biology. Trends Cell Biol..

[18] Heallen T (2013). Hippo signaling impedes adult heart regeneration. Development.

[19] Liu Q (2021). Suppressing Hippo signaling in the stem cell niche promotes skeletal muscle regeneration. Stem Cells.

[20] Feige JN, Auwerx J (2007). Transcriptional coregulators in the control of energy homeostasis. Trends Cell Biol..

[21] Finck BN, Kelly DP (2006). PGC-1 coactivators: inducible regulators of energy metabolism in health and disease. J. Clin. Invest..

[22] Bartrons R, Caro J (2007). Hypoxia, glucose metabolism and the Warburg's effect. J Bioenerg. Biomembr..

[23] Fothergill-Gilmore LA, Michels PA (1993). Evolution of glycolysis. Prog. Biophys. Mol. Biol..

[24] Murphy MP (2009). How mitochondria produce reactive oxygen species. Biochem. J..

[25] Kohen R, Nyska A (2002). Oxidation of biological systems: Oxidative stress phenomena, antioxidants, redox reactions, and methods for their quantification. Toxicol. Pathol..

[26] Krämer A, Green J, Pollard J, Tugendreich S (2014). Causal analysis approaches in ingenuity pathway analysis. Bioinformatics.

[27] Bartlett K, Eaton S (2004). Mitochondrial beta-oxidation. Eur. J. Biochem..

[28] Yang C (2014). Glutamine oxidation maintains the TCA cycle and cell survival during impaired mitochondrial pyruvate transport. Mol. Cell.

[29] Hargreaves M, Spriet LL (2020). Skeletal muscle energy metabolism during exercise. Nat. Metab..

[30] Kelley DE, Mandarino LJ (2000). Fuel selection in human skeletal muscle in insulin resistance: a reexamination. Diabetes.

[31] Chang HC, Guarente L (2014). SIRT1 and other sirtuins in metabolism. Trends Endocrinol. Metab..

[32] Landry J (2000). The silencing protein SIR2 and its homologs are NAD-dependent protein deacetylases. Proc. Natl. Acad. Sci. USA.

[33] Huang JY (2010). Mitochondrial sirtuins. Biochim. Biophys. Acta.

[34] Wang CH, Wei YH (2020). Roles of mitochondrial sirtuins in mitochondrial function, redox homeostasis, insulin resistance and type 2 diabetes. Int. J. Mol. Sci..

[35] Jing E (2011). Sirtuin-3 (Sirt3) regulates skeletal muscle metabolism and insulin signaling via altered mitochondrial oxidation and reactive oxygen species production. Proc. Natl. Acad. Sci. USA.

[36] Phielix E, Mensink M (2008). Type 2 diabetes mellitus and skeletal muscle metabolic function. Physiol. Behav..

[37] Sergi D (2019). Mitochondrial (dys)function and insulin resistance: From pathophysiological molecular mechanisms to the impact of diet. Front. Physiol..

[38] Sosnowska B (2017). The sirtuin family members SIRT1, SIRT3 and SIRT6: Their role in vascular biology and atherogenesis. Atherosclerosis.

[39] Rodgers JT (2005). Nutrient control of glucose homeostasis through a complex of PGC-1alpha and SIRT1. Nature.

[40] Lagouge M (2006). Resveratrol improves mitochondrial function and protects against metabolic disease by activating SIRT1 and PGC-1alpha. Cell.

[41] Deng Y (2016). Prostacyclin-producing human mesenchymal cells target H19 lncRNA to augment endogenous progenitor function in hindlimb ischaemia. Nat. Commun..

[42] Strober, W. Trypan blue exclusion test of cell viability. *Curr Protoc Immunol* **Appendix 3**, Appendix 3B (2001). 10.1002/0471142735.ima03bs21 18432654

[43] Chacko BK (2014). The bioenergetic health index: A new concept in mitochondrial translational research. Clin. Sci. (Lond.).

[44] Quiros PM, Goyal A, Jha P, Auwerx J (2017). Analysis of mtDNA/nDNA Ratio in Mice. Curr. Protoc. Mouse Biol..

