# Supplementary material for: Hippo pathway inhibition promotes metabolic adaptability and antioxidant response in myoblasts
Source: Sci Rep. 2023 Feb 8;13:2232. doi: 10.1038/s41598-023-29372-8 (PMC9908881; doi:10.1038/s41598-023-29372-8)
Supplement: Supplementary file 1 — Supplementary Information. [file 41598_2023_29372_MOESM1_ESM.pdf]

Hippo pathway inhibition promotes metabolic adaptability and antioxidant response in myoblasts

Qi Liu[1*], Su Pan[1], Pengyang Li[2], & Richard A. F. Dixon[1]

[1]Wafic Said Molecular Cardiology Research Laboratories, Texas Heart Institute, Houston, Texas, USA. [2]Division of Cardiology, Pauley Heart Center, Virginia Commonwealth University, Richmond, Virginia, USA.

[*]Qi Liu, PhD, Wafic Said Molecular Cardiology Research Laboratories, Texas Heart Institute, P.O. Box 20345, MC 2-255, Houston, Texas 77225-0345, USA. e-mail: QLiu@texasheart.org

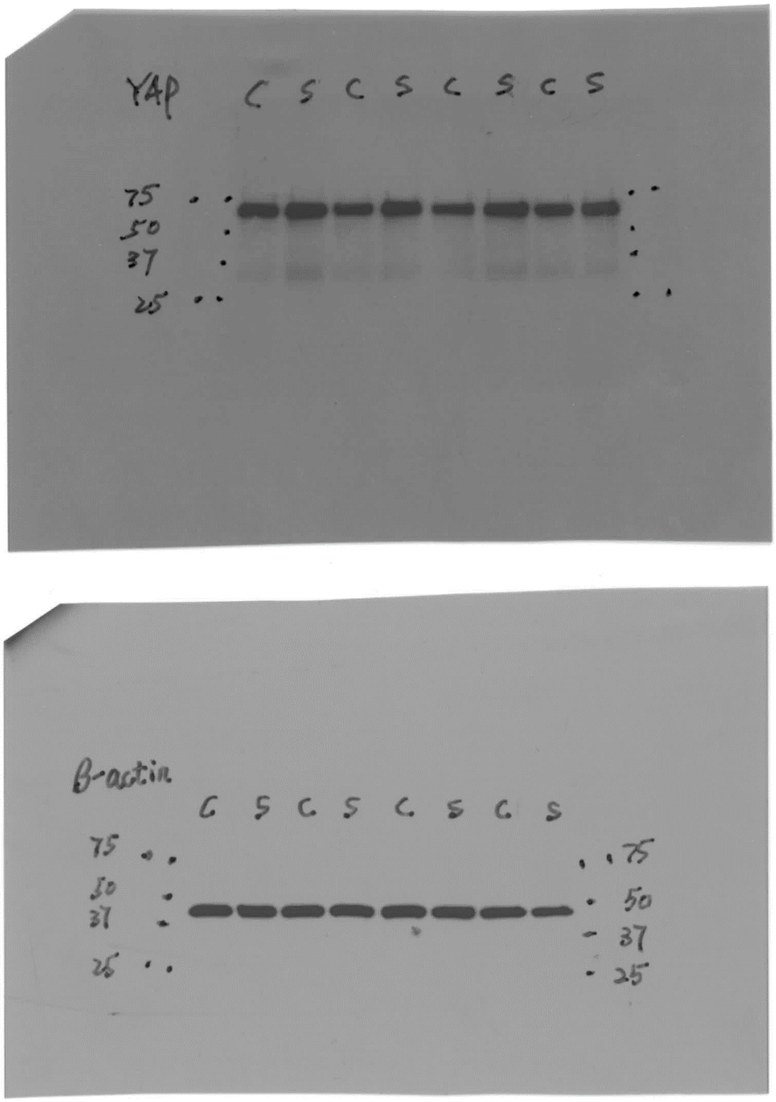

Supplementary Figure 1. Immunoblots with size marker indications. Full-length western blot images of total YAP and β-actin protein levels at 72 hours after siRNA transfection of C2C12 cells. β-actin was used as a loading control. The images are summaries of 4 independent siRNA transfection assays. C= negative control siRNA transfection, S=SAV1 siRNA transfection.

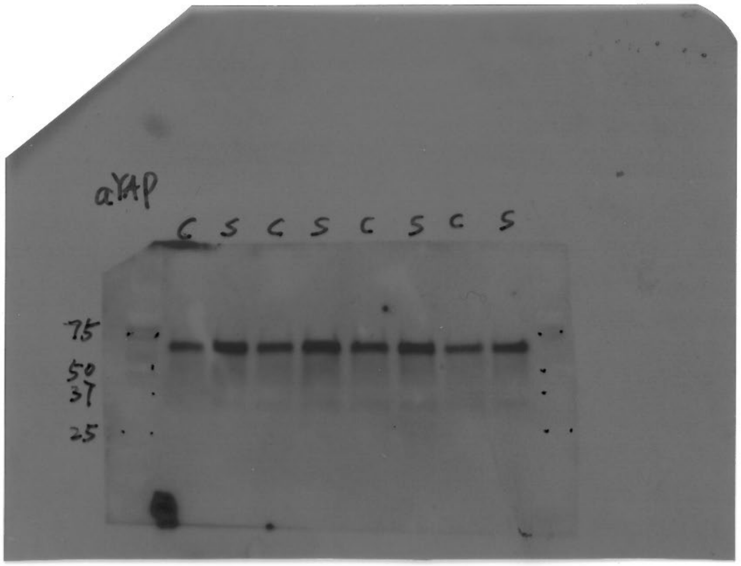

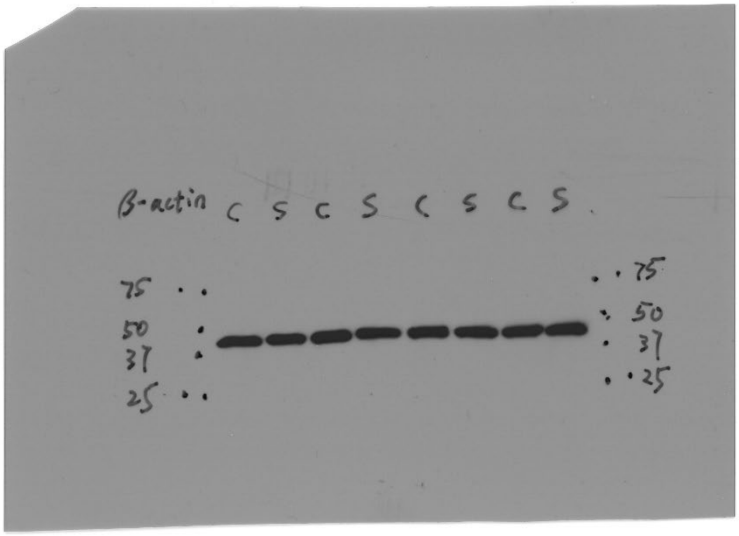

Supplementary Figure 2. Immunoblots with size marker indications. Full-length western blot images of the non-phosphorylated active form of YAP1 (aYAP) and β-actin protein levels at 72 hours after siRNA transfection of C2C12 cells. β-actin was used as a loading control. The images are summaries of 4 independent siRNA transfection assays. C= negative control siRNA transfection, S=SAV1 siRNA transfection.
